# 

**Published:** 1878-10

**Authors:** 


					﻿Whole Number.
cccci.
October, 1878,
Number 4,
Vol. XXXVII.
THE
CHICAGO
MEDIC AL
T ournal and Examiner
EDITORS:
WILLIAM H. BYFORD, A.M., M.D.
JAS. NEVINS HYDE, A.M., M.D. FERD. C. HOTZ, M.D
E. FLETCHER INCALS, M.D.
CHICAGO:
PUBLISHED BY THE CHICAGO MEDICAL PRESS ASSOCIATION.
188 Clark Street.
;4s“Read the Notice of this Journal among Advertisements.
				

